# Correlates, motivating factors, and barriers of engaging in regular self-reflection among public health students in higher education—A mixed methods approach

**DOI:** 10.3389/fpubh.2022.1023439

**Published:** 2022-11-03

**Authors:** Raymond Boon Tar Lim, Claire Gek Ling Tan, Kenneth Wee Beng Hoe, Cecilia Woon Chien Teng, Andre Matthias Müller, Julian Azfar, Suganthi Narayanasamy, Chee Hsiang Liow

**Affiliations:** Saw Swee Hock School of Public Health, National University of Singapore and National University Health System, Singapore, Singapore

**Keywords:** reflection, public health, pedagogy, curriculum, undergraduate, education, student engagement, active learning

## Abstract

Despite the wide implementation of self-reflection in higher education, the body of literature has predominantly focused on students from the clinical health sciences rather than public health. The objective of this study was to evaluate the correlates as well as to explore the motivating factors and barriers of engaging in regular self-reflection among public health students in higher education. We used a mixed methods approach (explanatory sequential design), comprising a cross-sectional survey (quantitative phase) followed by in-depth interviews (qualitative phase). We evaluated the association between reflection frequency as well as the perceptions and facilitators in reflection using the modified Breslow-Cox proportional hazards regression model. Prevalence ratio (PR) was generated. Thematic data analysis was carried out to analyse the qualitative data. Quantitative findings revealed being a regular reflector was positively associated with being more motivated to learn when one applied self-reflection (adjusted PR 1.60, 95% CI 1.17–2.20), the perception of being more prepared for a public health career in the future (adjusted PR 1.28, 95% CI 1.02–1.60), as well as being given enough opportunities to carry out self-reflection in the public health modules (adjusted PR 1.24, 95% CI 1.05–1.45). Qualitative findings revealed most students started their self-reflection mainly due to extrinsic factors such as institutional support, social support, teacher influence and environmental influence. Of these, the most prominent was teacher influence, indicating that they are key agents in promoting self-reflection. Students expressed that it would be important to cultivate intrinsic motivation to sustain their practice of self-reflection along the learning journey such as for the development of career-related professional skills. Other than intrinsic motivation, environmental influences were also important to promote continual reflection among students such as the availability of ample opportunities. Prominent barriers to address included external student factors such as the imbalanced power relationship between teacher and student, and internal student factors such as the perception that self-reflection was too cumbersome and time consuming.

## Introduction

Reflection, or self-reflection, in higher education was first brought into the limelight by the work of Dewey (1). This is defined as the self-observation and report of one's thoughts, desires, and feelings. It is a conscious cognitive procedure that relies on thinking, reasoning, and examining one's thoughts, feelings, and ideas (2). There are several benefits associated with the adoption of self-reflection. Self-reflection can improve learning (3, 4) and academic performance (5), and sets the foundation for lifelong learning (1, 6, 7). It can facilitate the integration of theory and practice (1, 3, 6, 7), which is especially invaluable given the shift toward competency-based education in the health sciences (6, 8). At a more personal level, self-reflection can also improve self-awareness (8), promote critical thinking (1, 5, 6, 9, 10) and problem-solving capabilities (5, 10). In view of this, self-reflection has been adopted in higher education training and accreditation standards in Europe and the United States of America, USA (11). For example, self-reflection has not only been widely implemented in medical (12), midwifery (1, 6), and nursing programmes (13), it has also been incorporated into the prerequisites for professional registration (1, 14).

Research on self-reflection has more than quadrupled in higher education in the past 20 years (15). Despite its wide implementation in higher education as well as the sizeable pool of empirical studies, there are a few gaps in the literature. First, the body of literature on self-reflection in health sciences higher education has predominantly focused on students from nursing and other clinical health sciences (10) but not public health. For example, self-reflection has been utilized as a pedagogical tool in various educational interventions in medicine (16–18), nursing (13, 19), pharmacy (7, 20), and dentistry (21). In contrast, studies focusing on public health students are sparse (22). Considering that public health education is dissimilar to clinical sciences, findings from studies available might not translate well to the public health context.

Second, the existing literature in higher education has largely focused on the development and evaluation of models for self-reflection. Commonly, authors proposed their own models for reflection or focused on the evaluation of such models in practice (6, 7, 16, 20, 23). For example, Lefroy et al. reported the use of video-assisted reflection using realist methodology to improve students' ability to self-reflect (23). Other types of studies focused on validating rubrics for the purposes of evaluating the quality and depth of students' reflections (17, 24). There was however a lack of studies to investigate the correlates, motivating factors and barriers to conducting regular self-reflection from the students' perspective. It would be important to elicit these to promote deep reflection in higher education. For example, while Chan et al. provided an overview of the challenges of encouraging reflection in higher education (15), motivating factors, which could offer valuable insights for future curriculum and programme improvement, were not reported.

Third, Deslandes reminds us of the importance of engaging with all stakeholders, especially students, who are arguably the most important stakeholder as the main beneficiaries of educational interventions (7). Students' voices are rarely, if ever, uniform, and their abilities to self-reflect also vary (4). It would thus be crucial to consider their perspectives in the development of reflection approaches (7, 13, 25). In addition, previous studies found that incorporating self-reflection into the curriculum by a “top-down” approach altered how students approached self-reflection (9, 23, 26), and may ultimately impact its effectiveness negatively (9, 23, 27). An example would be mandating the format of self-reflection after certain educational activities were carried out (19, 28). Additionally, there have only been a handful of studies that investigated students' motivations for self-reflection. One such study focused on developing and validating a tool to assess students' motivations (29), which was subsequently adapted to be more specific to medical students (30). Other studies have also reported students' motivations for engaging in self-reflection like having a greater learning interest (31), choice of topic, and prior positive experience (23). Since the perspectives of these students were often assessed after self-reflection was implemented using the “top-down” approach, they might not accurately represent students' motivations nor were they representative of the voices of students who were regularly engaging in self-reflection out of their own will. Therefore, this paper aimed to (i) assess the prevalence and correlates of engaging in regular self-reflection among public health students out of their own volition, and (ii) explore the motivating factors and barriers to engaging in regular self-reflection from the students' perspective.

## Methods

### Study design

We used a mixed methods approach, comprising a cross-sectional survey (quantitative phase) followed by in-depth interviews (IDIs; qualitative phase). An explanatory sequential design was used where the first phase involved the collection and analysis of quantitative data, while the subsequent qualitative phase built on these results to explain what they meant (32). Based on the quantitative results, participants were categorized according to their frequency of reflection of their own volition. Qualitative interviews were conducted sequentially to explore motivating factors among the frequent reflectors and barriers among the non-frequent reflectors. The aim of interview was to learn everything the student could share about the research topic from his or her world's view to gain insights and to better understand the student's perspective (33).

### Setting

The entire study took place in the Saw Swee Hock School of Public Health, National University of Singapore (NUS) which is the only national school of public health in Singapore. The school has been offering an undergraduate Minor in Public Health programme since 2013 and saw its inaugural cohort under the Second Major programme in 2021.

### Quantitative phase

The cross-sectional survey was conducted between August 2021 and October 2021. The inclusion criterion was that the participant was an undergraduate student enrolled in either the Public Health Minor or Public Health Second Major programme in NUS at the time of the study. Because of the COVID-19 outbreak during the study period, we could not conduct a face-to-face recruitment since all classes were online. Instead, an open invitation through email was sent to all existing students enrolled in the programmes by a staff who did not have any influential relationship with the potential participants. Students who were interested to participate would have to indicate that they had read and understood the nature of the study as well as consent to participating before they could proceed.

### Survey questionnaire

The questionnaire was available in English and the definition of self-reflection was provided (2). The questions included sociodemographic characteristics, frequency, perceptions, and facilitators in self-reflection using modified questions from the Self-Reflection and Insights Scale (34) and the Self-Awareness Outcomes Questionnaire (35). Based on the responses provided in the question “*How often do you perform self-reflection in your learning of public health modules on your own will?”* which used a five-point Likert scale ranging from “*None at all”* to “*All of the time”*, respondents were assessed on their frequency of performing self-reflection. The responses of “*None at all”* and “*Rarely”* were classified as infrequent self-reflectors, and the responses of “*Some of the time”*, “*Most of the time”*, and “*All of the time”* classified as frequent self-reflectors (34, 35). For each statement on the perceptions and facilitators in self-reflection, a five-point Likert scale was used (completely disagree, disagree, neutral, agree, completely agree). The scale was subsequently dichotomised into “*disagree/neutral”* (completely disagree, disagree, neutral) and “*agree”* (agree, completely agree) for meaningful analysis. To minimize social desirability biases, we (i) ensured the questionnaire was self-administered online, (ii) stressed the importance of responding honestly because their responses would be used for programme improvement, and (iii) ensured that the questionnaire was worded in a non-judgemental manner.

### Statistical analysis

We obtained the prevalence of participants who were frequent and infrequent reflectors. Bivariate analyses between their reflection frequency and each independent variable were carried out. Categorical variables were compared using chi-square test while continuous variables were compared using independent *t*-test. All sociodemographic characteristics were not associated with reflection frequency. We then evaluated the association between reflection frequency with each variable using the modified Breslow-Cox proportional hazards regression model to generate prevalence ratio (PR). This is a better alternative for the analysis of cross-sectional studies than logistic regression when the outcomes are common (>10% of the study population) (36). To identify the independent factors statistically, those with bivariate analysis of *p* ≤ 0.05 were selected for multivariable analysis. A backward stepwise approach was performed to obtain the adjusted PR (aPR) and 95% CI, where only variables with *p* ≤ 0.05 were included in the final model. All statistical analyses were performed using STATA version 15.0.

### Qualitative phase

Of the 164 participants who took the survey, 20 underwent IDIs in NUS from September 2021 to December 2021. Participants were asked to indicate in the survey questionnaire whether they were keen for the IDIs. Maximum variation sampling strategy was then used to recruit a purposive sample from diverse backgrounds, based on the criteria of sex, age, year of study and reflection frequency. The topic guide was pilot tested before study commencement and iteratively refined based on participants responses. It consisted of open-ended questions to explore and better understand the participants' reasons for their self-reflection behavior. Due to the ongoing COVID-19 pandemic, all interviews were held *via* video call. All participants had their earphones on during the interview or were alone, which provided privacy for the IDIs to be conducted. All interviews were conducted by the second and third authors who did not have any teacher-student relationship with the participants. The interviews were audio recorded with consent. We reached data saturation after 16 participants.

### Qualitative data analysis

The interviews were transcribed verbatim and checked for accuracy against the recordings. These were then imported into NVivo 11.0 and coded line-by-line. The first and third authors coded and analyzed the data in parallel, independently. Thematic data analysis was carried out, guided by the six-step procedure from Braun and Clarke (37). This involved multiple reading of the transcripts to get familiarized with the data. The initial codes were subsequently generated by the two authors independently before coming together to establish inter-coder reliability. This was achieved through discussing and resolving discrepancies in coding through discussion between the two authors. The codebook was continuously refined with additional codes emerging during the process. This occurred iteratively until inter-coder reliability was achieved at the ninth transcript. The finalized codebook was used to code the remaining transcripts. The codes were then categorized and condensed into preliminary subthemes and themes by the same two authors independently. Any discrepancy was again resolved by consensus.

### Ethics approval and participant consent

The study was approved by the NUS Learning and Analytics Committee on Ethics (approval reference code L2021-07-01). We obtained consent from all the participants of this study.

## Results

### Quantitative phase results

Out of 491 students to whom the email invitation was sent, a total of 179 responded. The participation rate was 36.6%. Non-participants did not differ significantly from participants in terms of sex, age, and year of study. “Busy” and “not interested” were the main reasons cited for non-participation. Of the 179 respondents, 15 were excluded from the analysis because of missing data in the key variables. The completion rate of the survey was 91.6%. Table 1 showed the survey participant characteristics. The prevalence of participants who were frequent reflectors was 73.8%. Of note, there was no statistical difference in sex, age, year of study, faculty/school, first major and type of public health programme enrolled between frequent and infrequent reflectors.

**Table 1:** Demographic characteristics of survey participants.

**Demographic characteristic**	**Total (*N* = 164)**	**Infrequent reflector**	**Frequent reflector**	***p*-value**
	***n* (%)**	**(*N* = 43) *n* (%)**	**(*N* = 121) *n* (%)**	
**Sex**				
Male	48 (29.3)	9 (20.9)	39 (32.2)	0.16
Female	116 (70.7)	34 (79.1)	82 (67.8)	
**Year of study**				
Junior (Year 1 and 2)	75 (45.7)	21 (48.8)	54 (44.6)	0.63
Senior (Year 3 and above)	89 (54.3)	22 (51.2)	67 (55.4)	
**Faculty/School**				
Arts and Social Sciences	23 (14.0)	7 (16.3)	16 (13.2)	0.78
Business	12 (7.3)	2 (4.7)	10 (8.3)	
Computing	3 (1.8)	0	3 (2.5)	
Design and Environment	3 (1.8)	1 (2.3)	2 (1.7)	
Engineering	26 (15.9)	6 (14.0)	20 (16.5)	
Science	89 (54.3)	26 (60.5)	63 (52.1)	
Others	8 (4.9)	1 (2.3)	7 (5.8)	
**First major**				
Chemistry	4 (2.4)	1 (2.3)	3 (2.5)	0.12
Communications and New Media	2 (1.2)	0	2 (1.7)	
Economics	7 (4.3)	0	7 (5.8)	
Food Science and Technology	7 (4.3)	0	7 (5.8)	
Life Sciences	68 (41.5)	22 (51.2)	46 (38.0)	
Pharmacy	7 (4.3)	1 (2.3)	6 (5.0)	
Psychology	8 (4.9)	4 (9.3)	4 (3.3)	
Social Work	2 (1.2)	1 (2.3)	1 (0.8)	
Sociology	2 (1.2)	1 (2.3)	1 (0.8)	
Others	57 (34.8)	13 (30.2)	44 (36.4)	
**Public health programme enrolled**				
Minor	147 (89.6)	36 (83.7)	111 (91.7)	0.14
Second Major	17 (10.4)	7 (16.3)	10 (8.3)	
**Mean age in years (Standard deviation)**	21.3 (1.6)	20.9 (1.5)	21.4 (1.6)	0.13

Table 2 shows the perceptions and facilitators in self-reflection in public health modules by reflection frequency. Frequent reflectors reported more positive perceptions and greater levels of facilitators in self-reflection than their counterparts.

**Table 2:** Perceptions and facilitators in self-reflection in public health modules by reflection frequency.

**Factor**	**Total (*N* = 164) *n* (%)**	**Infrequent reflector (*N* = 43) *n* (%)**	**Frequent reflector (*N* = 121) *n* (%)**	***p*-value**
**Perceptions of self-reflection**				
1. I think that self-reflection is important in learning.				
Disagree/Neutral	15 (9.1)	12 (27.9)	3 (2.5)	< 0.001
Agree	149 (90.9)	31 (72.1)	118 (97.5)	
2. I am willing to apply self-reflection in learning.				
Disagree/Neutral	21 (12.8)	15 (34.9)	6 (5.0)	< 0.001
Agree	143 (87.2)	28 (65.1)	115 (95.0)	
3. I am more motivated to learn when I apply self-reflection.				
Disagree/Neutral	47 (28.7)	25 (58.1)	22 (18.2)	< 0.001
Agree	117 (71.3)	18 (41.9)	99 (81.8)	
4. I believe that my module grade will improve when I apply self-reflection.				
Disagree/Neutral	55 (33.5)	23 (53.5)	32 (26.4)	0.001
Agree	109 (66.5)	20 (46.5)	89 (73.6)	
5. I understand the module material better if I perform self-reflection.				
Disagree/Neutral	43 (26.2)	20 (46.5)	23 (19.0)	< 0.001
Agree	121 (73.8)	23 (53.5)	98 (81.0)	
6. I am better able to apply the module concepts taught if I perform self-reflection.				
Disagree/Neutral	41 (25.0)	21 (48.8)	20 (16.5)	< 0.001
Agree	123 (75.0)	22 (51.2)	101 (83.5)	
7. Self-reflection prepares me for a public health career in the future.				
Disagree/Neutral	66 (40.2)	29 (67.4)	37 (30.6)	< 0.001
Agree	98 (59.8)	14 (32.6)	84 (69.4)	
**Facilitators in self-reflection**
8. I have been given enough opportunities to carry out self-reflection in the public health modules.				
Disagree/Neutral	98 (59.8)	35 (81.4)	63 (52.1)	0.001
Agree	66 (40.2)	8 (18.6)	58 (47.9)	
9. I know how to perform self-reflection.				
Disagree/Neutral	88 (53.7)	34 (79.1)	54 (44.6)	< 0.001
Agree	76 (46.3)	9 (20.9)	67 (55.4)	
10. I have the confidence to perform self-reflection by myself.				
Disagree/Neutral	88 (53.7)	35 (81.4)	53 (43.8)	< 0.001
Agree	76 (46.3)	8 (18.6)	68 (56.2)	
11. I will perform self-reflection if it is part of the module assessment or assignment.				
Disagree/Neutral	21 (12.8)	6 (14.0)	15 (12.4)	0.79
Agree	143 (87.2)	37 (86.0)	106 (87.6)	
12. I am more willing to perform self-reflection if the lecturer of the module advocates for it.				
Disagree/Neutral	43 (26.2)	14 (32.6)	29 (24.0)	0.27
Agree	121 (73.8)	29 (67.4)	92 (76.0)	
13. I am more willing to perform self-reflection if the lecturer of the module teaches us the skills on it.				
Disagree/Neutral	19 (11.6)	7 (16.3)	12 (9.9)	0.26
Agree	145 (88.4)	36 (83.7)	109 (90.1)	

Table 3 shows the crude and adjusted prevalence ratio (PR) of correlates of self-reflection frequency in public health modules. The prevalence of frequent reflectors who agreed to the statement, “*I am more motivated to learn when I apply self-reflection”* was higher than those who disagreed or were neutral (PR 1.78; 95% CI: 1.30–2.44). Compared to those who disagreed or were neutral to the statement, “*Self-reflection prepares me for a public health career in the future”*, the prevalence of frequent reflectors was higher in those who agreed (PR 1.50; 95% CI: 1.19–1.89). Similarly, the prevalence of frequent reflectors who agreed to the statement, “*I have been given enough opportunities to carry out self-reflection in the public health modules”* was higher compared to those who disagreed or were neutral (PR 1.37; 95% CI: 1.15–1.63). Multivariable analyses showed that being a frequent reflector was positively associated with being more motivated to learn when one applied self-reflection (aPR 1.60, 95% CI 1.17–2.20), the perception of being more prepared for a public health career in the future (aPR 1.28, 95% CI 1.02–1.60), and being given enough opportunities to carry out self-reflection in the public health modules (aPR 1.24, 95% CI 1.05–1.45).

**Table 3:** Crude and adjusted prevalence ratio (PR) of correlates of self-reflection frequency in public health modules.

**Factor**	**Crude PR (95% CI)**	***p*-value**	**Adjusted PR (95% CI)**	***p*-value**
**Perceptions of self-reflection**				
1. I am more motivated to learn when I apply self-reflection.				
Disagree/Neutral		Reference		
Agree	1.78 (1.30–2.44)	< 0.001	1.60 (1.17–2.20)	0.003
2. Self-reflection prepares me for a public health career in the future.				
Disagree/Neutral		Reference		
Agree	1.50 (1.19–1.89)	0.001	1.28 (1.02–1.60)	0.03
**Facilitator in self-reflection**				
3. I have been given enough opportunities to carry out self-reflection in the public health modules.				
Disagree/Neutral		Reference		
Agree	1.37 (1.15–1.63)	< 0.001	1.24 (1.05–1.45)	0.009

### Qualitative phase results

**Figure 1:**
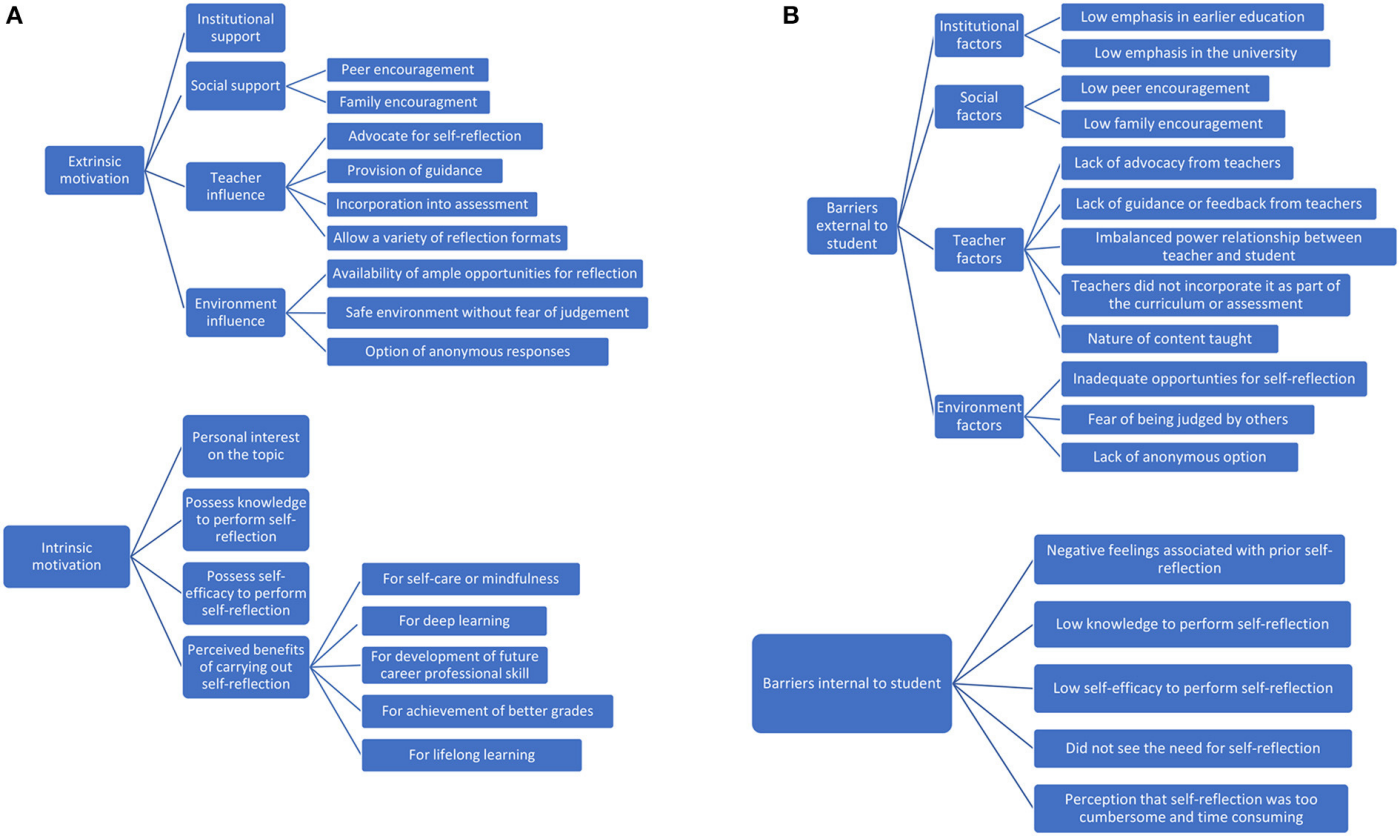
**(A)** Themes and subthemes of the motivating factors to carrying out regular self-reflection among students. **(B)** Themes and subthemes of the barriers to carrying out regular self-reflection among students.

Table 4 showed the IDI participant characteristics. Figure 1A outlines the themes and subthemes of the motivating factors to carrying out regular self-reflection among students. Appendix 1(A) shows the corresponding illustrative quotes. There were both extrinsic and intrinsic motivation factors. Students started their self-reflection journey mainly due to extrinsic factors such as institutional support, social support, teacher influence and environmental influence. Of these, the most prominent was teacher influence, indicating that they are key agents in promoting self-reflection. For example, it was common to hear comments like, “*I think self-reflection happened in my education because of the teachers who were interested in bringing this to the classroom. It wasn't like a standardized or intuitive thing to do in classroom I would say. It wasn't a norm but there were teachers who brought this in which I really appreciated. If not, I would have never realized the importance of it or practiced it till today.”* (S3).

**Table 4:** Demographic characteristics of participants for in depth interviews.

**Demographic characteristic**	**Total (*N* = 20) *n* (%)**	**Infrequent reflector (*N* = 5) *n* (%)**	**Frequent reflector (N = 15) *n* (%)**
**Sex**			
Male	6 (30.0)	1 (20.0)	5 (33.3)
Female	14 (70.0)	4 (80.0)	10 (66.7)
**Year of study**			
Junior (Year 1 and 2)	9 (45.0)	2 (40.0)	7 (46.7)
Senior (Year 3 and above)	11 (55.0)	3 (60.0)	8 (53.3)
**Faculty/School**			
Arts and Social Sciences	3 (15.0)	1 (20.0)	2 (13.3)
Engineering	1 (5.0)	0	1 (6.7)
Science	16 (80.0)	4 (80.0)	12 (80.0)
**First major**			
Chemistry	1 (5.0)	0	1 (6.7)
Food Science and Technology	2 (10.0)	0	2 (13.3)
Life Sciences	13 (65.0)	4 (80.0)	9 (60.0)
Psychology	1 (5.0)	0	1 (6.7)
Sociology	1 (5.0)	1 (20.0)	0
Others	2 (10.0)	0	2 (13.3)
**Public health programme enrolled**			
Minor	15 (75.0)	4 (80.0)	11 (73.3)
Second Major	5 (25.0)	1 (20.0)	4 (26.7)
**Mean age in years (Standard deviation)**	21.9 (1.48)	21.4 (0.55)	22.1 (1.67)

Students expressed four aspects relating to the roles of teachers in facilitating frequent reflection. Teachers could advocate for self-reflection, “*...Yeah. I think most of the professors are very supportive because they really encourage us to formulate our own thoughts and like we can – they even encourage us to share with other people.”* (S40), provide guidance, “*Oh, one thing that the Prof did was helpful was he shared a reflection paper, he shared the papers of those that did well to the class. So, we kind of know like what he is looking for.”* (S7), incorporate reflection into their assessments, “*I guess it is like those lecturers who incorporate self-reflection into the assessments. Yeah, like there is a small percentage of marks that is attached to it. And then like we the students were encouraged to do it*.” (S38), and allow a variety of reflection formats, “*I appreciate there should be various formats of reflection that we could choose from – maybe because when compared to journaling, if there is oral discussion, I get to hear inputs of other people. For journaling it is mainly reflecting on what I feel, what my own thoughts are.”* (S12). The next prominent extrinsic motivation factor was social support. Other than peers, family encouragement was touted as another important motivating factor for initiation of reflection, “*It was my dad who first encouraged me to do it. He shared with me how he practiced self-reflection back in his university days and now at the workplace...that kind of prompted me to start.”* (S6).

Students expressed that it would be important to cultivate intrinsic motivation to sustain their practice of self-reflection along the learning journey. For example, it was common to hear comments like, “*While teachers are important to help us start our self-reflection journey in learning, at the end of the day, I feel intrinsic motivation is what continues to drive me and my peers, to apply this in my current university education, and in the future for my career. Without having such inherent motivation or goals, I think it is hard to sustain this given the various commitments we have.”* (S12). With regards to intrinsic motivation, students expressed that the perceived benefits of carrying out reflection was most crucial to them. There were five such perceived benefits. This included self-care or mindfulness, “*I think it helps me to be like more mindful. And that I actually practice what I learn. Yeah, so I don't like preach about it but to actually actively and apply it to my life.”* (S2), deep learning, “*I think yes, because especially for public health, I mean obviously like theoretical knowledge is one part of it, but if you want to go into the deeper meaning of it, you have to break down the public health problem and reflect in-depth. And based on how you break down, you tend to ideate solutions to solve the problems.”* (S13), development of career-related professional skills, “*I would say especially for policies, public health requires an in-depth self-reflection, not only from the student's part but definitely through years and years of seeing public health emergencies. Self-reflection is definitely exercised by public health policy makers. So, I think it is a good trait as an aspiring public health policy maker for me to bring forward in the future.”* (S37), achievement of better grades, “*Because self-reflection is necessary if you want to do better in the second group project, you will need to look back on your first project, like how it was graded? Could I have done better, and if so, how?”* (S21), and lifelong learning, “*... I mentioned that self-reflection enables me to keep track of my goals and objectives... I think on a personal level, it makes my learning more fulfilled, it motivates me to continue learning in my life. Because by doing that, I could manage my thoughts better, and to consolidate my thinking better.”* (S17).

Other than intrinsic motivation, environmental influences were also important to promote continual reflection. It was common to hear comments like, “*Having a long-term goal and the motivation is good, but sometimes it really helps if the learning environment we are in kind of nudges us into reflection, if not at least encourages us to do that.”* (S1). Students described three aspects of the environment that could facilitate this. They included availability of ample opportunities, “*I think there needs to be enough opportunities for us to reflect. It can be just simple things like quizzes. What's important is that there needs to self-reflection opportunities throughout the semester. Because having that self-reflection or recalling opportunities along the way have helped me significantly.”* (S17), promoting a safe environment without fear of judgement, “*I think the main point is being in a safe community. A community where you can share your thoughts freely and they don't judge you for it. Yeah, so when it comes to that, what I mean is by judging in terms of like the responses which I received when I say my thoughts. So, it is not just verbal but facial expressions, the body language too, and not being judged for it.”* (S38), and allowing anonymous responses, “*I feel like it is important to be anonymous, then people might be more willing to sharing. Because if our names are there, some people might feel embarrassed to share what their thoughts are. But if it is anonymous, then we won't really be that afraid to share our thoughts and reply to other people....”* (S40).

Figure 1B outlines the themes and subthemes of the barriers to carrying out regular self-reflection among students. Appendix 1(B) shows the corresponding illustrative quotes. Other than factors internal to students, external elements such as institutional, social support, teacher and environmental factors were barriers to carrying out regular self-reflection among students. An example of a recurring teacher-related barrier was the imbalanced power relationship between teacher and student, “*For me, I don't feel comfortable in reflecting or revealing what I really think. Because I feel like my lecturer has some sort of authority over the content he or she is sharing. So, I feel it will be not very nice for me to challenge him or her, even though when I have my doubts or when I think otherwise.”* (S14).

For internal factors, one recurring factor was the perception that self-reflection was too cumbersome and time consuming, “*I feel that writing all these reflective essays is very cumbersome, I will have to spend a lot of time on it and then instead of it being like an opportunity to reflect, it becomes more of like a chore. Yeah... as university students, we have like time constrains and if you compare us with other graduates in other countries, I will say that we don't have much time.”* (S3).

## Discussion

To the best of our knowledge, this is the first mixed methods study to evaluate the correlates as well as to explore the motivating factors and barriers of engaging in regular self-reflection among public health students in higher education. Being a regular reflector was positively associated with being more motivated to learn when one applied self-reflection, the perception of being more prepared for a public health career in the future, as well as being given enough opportunities to carry out self-reflection in public health modules. Qualitative findings revealed students started their self-reflection journey mainly due to extrinsic factors such as institutional support, social support, teacher influence and environmental influence. Of these, the most prominent was teacher influence. Students expressed that it would be important to cultivate intrinsic motivation to sustain their practice of self-reflection along the learning journey such as for development of career-related professional skills. Other than intrinsic motivation, environmental influences were also important to promote continual reflection among students such as the availability of ample opportunities. Prominent barriers to address included external student factors such as the imbalanced power relationship between teacher and student as well as internal student factors such as the perception that self-reflection was too cumbersome and time consuming.

This study showed a strong positive correlation between motivation to learn and engaging in regular reflection. This was similar to other studies where Sobral (38) reported a moderate correlation between motivation and reflection in learning (*r* 0.44) among medical students in Brazil, as well as Hsin-Hui Wang et al. (39) who reported self-reflection had a strong and direct relation to students' learning motivation (β = 0.86) among college students in Taiwan. Although the exact understanding of motivation to learning continues to evolve, it could be described for practical purposes as either extrinsic or intrinsic. Intrinsic motivation pertains to activities done “for one's own sake” or for their inherent interest and enjoyment, while extrinsic motivation comes from outside the individual (40). Students initiated self-reflection mainly due to extrinsic factors, of which the most prominent was teacher influence, indicating that they are key agents in promoting self-reflection. Teachers have an important role in being catalysts of change at the institutional level (41) in promoting a culture of reflective practice. This may entail teacher training and sharing of practices to instill confidence in facilitating and practicing reflection. One way to achieve this is through peer mentorship, where new teachers are mentored by their senior counterparts in using reflective activities in their teaching (42). At the classroom-level, teachers can encourage students to reflect on, analyse, evaluate, and improve their own learning. When done appropriately and effectively, these not only improve education quality but also convey institutional commitment in supporting reflective learning.

Students expressed that it would be important to cultivate intrinsic motivation to sustain their practice of self-reflection along the learning journey. Developing intrinsic motivation is key to developing autonomous learners who have acquired strong reflective thinking skills (43). Autonomous learners take responsibility and control over their own learning and can motivate themselves throughout the learning process. De la Croix adds that motivation is what makes students turn reflection into a lifelong practice (12). One intrinsic motivation from our quantitative and qualitative findings is in preparing for a future public health career. The process of self-discovery and awareness, and the development of a professional identity require self-reflection skills (44). Reflection connects new experiences with existing knowledge and skills in relation to the student's profession, helps students to make sense of their learning experiences, and increases their confidence in career-related decision-making. Having a strong professional identity contributes to individual commitment and career success (44). It is thus vital to incorporate learning activities that foster ethical and reflective practice in professional degree programmes.

However, for students to become regular reflective learners, extrinsic motivational factors are still needed as highlighted by our qualitative findings. While higher motivation yielded higher autonomy scores, many autonomous learners still felt unmotivated to complete tasks outside of the classroom (45). Therefore, it is imperative to have continual extrinsic motivation (46). This follows that if reflective learning is to be incorporated, then providing ample opportunities for self-reflection is crucial. When incorporating reflection practices into the curriculum, it is important to consider introducing it at the programme or degree level rather than in individual disparate courses. For example, reflective tasks should be scaffolded into the curriculum with ample opportunities for formative feedback and summative assessment to encourage critical thinking.

Several barriers were also identified in this study. To promote self-learning in students, steps must be taken to address them. These factors are often interconnected and remind us of the need to promote regular self-reflection across all levels from students to teachers and even educational institutions. One such teacher factor identified was the imbalanced power relationship between teacher and student. In general, the power distance between teacher and student is much greater in Asia compared to that in Europe and USA due to differences in sociocultural norms (47). This deserves attention because it strongly influences teacher-student relationships, students' motivation to learn, and learning outcomes (48). Teachers should seek to share this power with students to promote regular reflection. To achieve this, teachers could model the desired self-reflective behavior by first sharing their own reflective thoughts to inculcate this norm in their classrooms. This would then create a conducive environment so that students would be willing to share their reflective thoughts freely and openly too.

At the student level, one such factor was the perception that self-reflection was too cumbersome and time consuming. This was similar to other studies where students tended to perceive reflections as an additional burden on top of their workload (49, 50). These perceptions could negatively affect their motivation and engagement in regular reflection (51). If students do not regard self-reflection to be relevant or beneficial to their learning, they will treat it as a chore. If these negative perceptions are allowed to persist, it can potentially lead to half-hearted or unsustainable self-reflection behavior (51). This was reflected in our quantitative findings where infrequent reflectors reported more negative and lower levels of facilitators in self-reflection compared to frequent reflectors. As a start, it would be crucial to change the perceptions of these students. One way to do this is to appoint student ambassadors or champions who are regular reflectors. They could be trained by teachers or the institution to influence or facilitate reflection effectively among peers. If student ambassadors or champions work collaboratively with their peers, it might be easier to forge meaningful connections and to modify their perceptions.

### Implications for future studies

Based on this study, future researchers could assess the specific types of self-reflection formats that would have an impact on improving students' competencies for the public health profession as well as determine whether these are top-down or bottom-up. Various reflective formats like journal, focus group discussion, photovoice, and narrative reflective practice have been used as pedagogical tools in the public health curriculum (52, 53). While Artioli et al. conducted qualitative meta-synthesis on the use of reflective writing on health professionals, focusing on one type of reflection format is insufficient to determine the impacts of self-reflection in public health higher education (53). There is a need for future studies to identify the broad range of self-reflection formats available in public health education, and to assess whether they occur out of students' own volition (i.e., whether they are top-down or bottom-up) because that might have an implication on improving students' competencies for the profession.

### Strengths and limitations

There were several strengths and limitations of our current study. One strength was that the mixed methods approach enabled triangulation as some correlates in the quantitative analysis were also recurrent themes in the qualitative analysis. The qualitative findings enabled us to have an in-depth understanding of the self-reflection behavior among public health students in higher education. Another strength was that data saturation was reached for the qualitative analysis. Nevertheless, there were also a few limitations. First, during the qualitative phase, we did not show the transcript to the participants to confirm whether their responses had been accurately documented. However, the interviewers mitigated this by regularly paraphrasing and “checking back” with the participants to ascertain the veracity of their responses. Second, we could not exclude social desirability bias since data was self-reported. Nevertheless, we have described in the Methods section the steps which have been taken to reduce this bias. Third, causal relationships cannot be inferred from the cross-sectional study (quantitative component).

## Conclusion

To conclude, engaging in regular self-reflection was positively associated with being more motivated to learn when one applied self-reflection, the perception of being more prepared for a public health career in the future, as well as being given enough opportunities to carry out self-reflection in the public health modules. Students started their self-reflection journey mainly due to extrinsic factors such as institutional support, social support, teacher influence and environmental influence. Of these, teacher influence was a predominant factor. Students expressed that it would be important to cultivate intrinsic motivation to sustain their practice of self-reflection along the learning journey such as for development of career-related professional skills. Other than intrinsic motivation, environmental influences were also important to promote continual reflection among students such as provision of ample opportunities. Prominent barriers to address included external student factors such as the imbalanced power relationship between teacher and student as well as internal student factors such as the perception that self-reflection was too cumbersome and time consuming.

## Data availability statement

The original contributions presented in the study are included in the article/Supplementary material, further inquiries can be directed to the corresponding author/s.

## Ethics statement

The studies involving human participants were reviewed and approved by the National University of Singapore Learning and Analytics Committee on Ethics. The patients/participants provided their written informed consent to participate in this study.

## Author contributions

All authors listed have made a substantial, direct, and intellectual contribution to the work and approved it for publication.

## Funding

The Saw Swee Hock School of Public Health, National University of Singapore will cover the Open Access publication fee.

## Conflict of interest

The authors declare that the research was conducted in the absence of any commercial or financial relationships that could be construed as a potential conflict of interest.

## Publisher's note

All claims expressed in this article are solely those of the authors and do not necessarily represent those of their affiliated organizations, or those of the publisher, the editors and the reviewers. Any product that may be evaluated in this article, or claim that may be made by its manufacturer, is not guaranteed or endorsed by the publisher.

## Abbreviations

IDIs: In-Depth Interviews
NUS: National University of Singapore
PR: Prevalence Ratio
USA: United States of America.
