# Supplementary material for: Correlates, motivating factors, and barriers of engaging in regular self-reflection among public health students in higher education—A mixed methods approach
Source: Front Public Health. 2022 Nov 3;10:1023439. doi: 10.3389/fpubh.2022.1023439 (PMC9670312; doi:10.3389/fpubh.2022.1023439)
Supplement: Supplementary file 1 [file Table_1.DOCX]

**Appendix 1(A): Illustrative quotes for the themes and subthemes on the motivating factors to carrying out regular self-reflection among students**

| **Theme** | **Subtheme** | **Sub subtheme** | **Illustrative quotes** |
| --- | --- | --- | --- |
| Extrinsic motivation | Institutional support | - | “...yeah, I definitely think there has been a lot of encouragement from the institutional level, which made me eventually self-reflect on the content.” (S6) |
|  | Social support | Peer encouragement | "I have friends around me who also self-reflect. So that is like a strong motivator for me to do it too. Because I see that it really helps my friends to get through the day, and also to make improvements to small aspects of their lives...” (S17)  “I often need to rely on others to pinpoint it out, yeah so to a certain extent, I guess that is why I am quite thankful with the peer feedback we had in the group. It was quite like – so far it has been quite helpful, like yeah qualitatively.” (S38)  "Like when we have a group discussion, yeah it will definitely encourage me to participate in discussion more. Every time I do, I always learn new things that I didn’t really think before or didn’t really know before. I guess peer learning helps as each of us come from different background." (S2) |
|  |  | Family encouragement | “It was my dad who first encouraged me to do it. He shared with me how he practised self-reflection back in his university days and now at the workplace...that kind of prompted me to start.” (S6)  “I remembered it was my dad who inspired me to start my personal reflection. My dad was sharing with me how he would reflect and jot down his thoughts in his phone like a journal. So yeah, that kind of motivated me to do it as well.” (S17) |
|  | Teacher influence | Advocate for self-reflection | “I find myself being able to discuss what my thoughts are more, reflecting more. Yeah..., definitely. I feel like the public health lecturers are more encouraging, for the students to engage in self-reflection, as compared to my first major. And I think it is because of the whole structure of public health, and how public health is like.” (S12)  “... Yeah. I think most of the professors are very supportive because they really encourage us to formulate our own thoughts and like we can – they even encourage us to share with other people.” (S40)  " Yeah. I guess for those lecturers that are like quite candid in their responses, or even when teaching the concepts, they share their own thoughts or personal stories. Yeah so, I think it is a lot more relatable. This also kind of encourages me to also self-reflect...." (S38) |
|  |  | Provision of guidance | "Oh, one thing that the Prof did was helpful was he shared a reflection paper, he shared the papers of those that did well to the class. So, we kind of know like what he is looking for." (S7)  "I mean it will be good for the lecturers... like perhaps prepare a type of document for self-reflection for the students. Like at the end of the lesson, it will be quite useful for students in general. I mean the lecturers or professors who are willing to provide more guidance on what to write, or perhaps share with us what are the food for thought, this would encourage us to self-reflect. " (S37) |
|  |  | Incorporation into assessment | “I think if it wasn’t assessed, then people would be less likely to do it. So, I think there needs to be a sort of an assessment. For example, if the lecturer makes this into an assessment and then allocate weightage of marks to it like maybe 5% of the total grading of the module, I think that it will be more useful, so people will be like, oh I have to do it then" (S17)  "I guess it is like those lecturers who incorporate self-reflection into the assessments. Yeah, like there is a small percentage of marks that is attached to it. And then like we the students were encouraged to do it." (S38) |
|  |  | Allow a variety of reflection formats | "I appreciate there should be various formats of reflection that we could choose from – maybe because when compared to journaling, if there is oral discussion, I get to hear inputs of other people. For journaling it is mainly reflecting on what I feel, what my own thoughts are." (S12)  "It is important to have both formal and informal settings for reflection. And I think that platform of Twitter, really helped me to get into the mode of self-reflection because it wasn’t really a serious platform like say a forum, although that will also be okay, but I think, it really helped me to process whatever I learned.” (S6) |
|  | Environment influence | Availability of ample opportunities for reflection | "I think there needs to be enough opportunities for us to reflect. It can be just simple things like quizzes. What’s important is that there needs to self-reflection opportunities throughout the semester. Because having that self-reflection or recalling opportunities along the way have helped me significantly." (S17)  "The environment must give us time and space to do so. I think for example the public health mods or the GE mods, they provide chances for us to do that. Yeah, then there is like more attention paid to it." (S38) |
|  |  | Safe environment without fear of judgement | "I think the main point is being in a safe community. A community where you can share your thoughts freely and they don’t judge you for it. Yeah, so when it comes to that, what I mean is by judging in terms of like the responses which I received when I say my thoughts. So, it is not just verbal but facial expressions, the body language too, and not being judged for it.” (S38)  "Yeah, I think the safe environment aspect was definitely there. But it was also conducive because in the modules that I have taken, people will reply to the discussion, and engage in further discussions. The lecturers and professors also heavily emphasised for everyone to be respectful and respecting other student’s views, so I think it laid the ground for a safe discussion.” (S6) |
|  |  | Option of anonymous responses | "Ensuring anonymity helps. Because nobody knows. So, you can freely type and then I guess people will be more genuine and sincere in their responses." (S38)  "I feel like it is important to be anonymous, then people might be more willing to sharing. Because if our names are there, some people might feel embarrassed to share what their thoughts are. But if it is anonymous, then we won’t really be that afraid to share our thoughts and reply to other people...." (S40) |
| Intrinsic motivation | Personal interest on the topic | - | “Like if the area of the study is my interest right, then I will be like more passionate to do the self-reflection. But if it is an area which I am not interested in and I am forced to do self-reflection right, then maybe I won’t really enjoy it as much...." (S40)  "I think more than the rapport, but also the topic. And the way the question was phrased for self-reflection. But most importantly it is the topic that must naturally interest a lot of students." (S6)  "I think it depends on how invested I am in the particular situation or topic. So maybe for example in like public health, I am more interested in a lot of diseases, so I will really think about why certain interventions wouldn’t work even though I feel like it is not too bad of an intervention. But why the success rate is low." (S12) |
|  | Possess knowledge to perform self-reflection | - | "Hmm, I would think that I have the knowledge to do it. Like – like I know which mode of self-reflection will work for me, erm like sometimes it is just thinking through it or sometimes it is just penning down my thoughts in my notebook." (S6) |
|  | Possess self-efficacy to perform self-reflection | - | " I think having that confidence to do reflection is quite important because this means you believe in yourself that you can carry out self-reflection. So, you are more motivated to conduct self-reflection if you have that." (S40) |
|  | Perceived benefits of carrying out self-reflection | For self-care or mindfulness | "I guess to pay attention to mental health, so I guess in terms of self-reflection, something that I thought was related was my mindfulness. I guess when you are mindful about something, that is you also intentionally reflecting on different things. Yeah, like mindful to surroundings, of the things that you are currently doing or learning" (S38)  "I think it helps me to be like more mindful. And that I actually practise what I learn. Yeah, so I don’t like preach about it but to actually actively and apply it to my life." (S2)  "... it important for self-care I suppose. Because I have been able to feel less stressed out, I guess? Because I have been able to manage my thoughts better, and to manage my life better. So, I feel that on a personal level, that it has made some sort of difference in my life." (S17) |
|  |  | For deep learning | "I think yes, because especially for public health, I mean obviously like theoretical knowledge is one part of it, but if you want to go into the deeper meaning of it, you have to break down the public health problem and reflect in-depth. And based on how you break down, you tend to ideate solutions to solve the problems." (S13)  "I think it is important because there is a lot of content. Public health is not only content heavy but also a lot of application, so it requires a lot of understanding and I think having a reflection helps you to consolidate your information and also to help you better understand the concept so that you can apply it in the future...” (S17)  "Yeah, and I also feel like self-reflection is quite important, because if there is no self-reflection, you won’t understand the concepts taught. You won’t be able to relate to it, it will just be like another piece of information. So, self-reflection enables me to consolidate the thought processes, like how public health policies were drafted out, and then yeah like when you create policies, you need to be careful of certain loopholes and certain assumptions you make. For example, did you neglect a certain group of people in your policies? So, in terms of self-reflection that was quite helpful because it enables me to understand the concepts taught at a deeper level." (S38) |
|  |  | For development of future career professional skill | "I think in terms of career, it also helps. Because when you have the habit of self-reflecting, then it will be useful for the future when you work. I do think it is important to have self-reflection as a skill in the future for work...Hmm… for example when you take on a project from work and you hit certain obstacles, it would be important to reflect on what you have done and be able to identify which area needs to be addressed and be improved on. So, I think maybe in that aspect, self-reflection is an important skill to develop for one’s future career." (S17)  "I would say especially for policies, public health requires an in-depth self-reflection, not only from the student’s part but definitely through years and years of seeing public health emergencies. Self-reflection is definitely exercised by public health policy makers. So, I think it is a good trait as an aspiring public health policy maker for me to bring forward in the future." (S37) |
|  |  | For achievement of better grades | " Because self-reflection is necessary if you want to do better in the second group project, you will need to look back on your first project, like how it was graded? Could I have done better, and if so, how?" (S21)  "... The grades...Yeah, this is an important motivating factor for self-reflection if we feel like we want to do well for this module, so we will then carry out self-reflection." (S7) |
|  |  | For lifelong learning | "Hmm, I think self-reflection motivates me to learn better, especially for lifelong learning. You never know what you are going to encounter later in life. When I meet a failure or when I feel stuck in my learning, I would take a step back and look at the bigger picture and reflect on what could have been done better, and how I could move on from there." (S12)  "... I mentioned that self-reflection enables me to keep track of my goals and objectives... I think on a personal level, it makes my learning more fulfilled, it motivates me to continue learning in my life. Because by doing that, I could manage my thoughts better, and to consolidate my thinking better." (S17) |

**Appendix 1(B): Illustrative quotes for the themes and subthemes on the barriers to carrying out regular self-reflection among students**

| **Theme** | **Subtheme** | **Sub subtheme** | **Illustrative quotes** |
| --- | --- | --- | --- |
| Barriers external to student | Institutional factors | Low emphasis in earlier education | "Yeah, in secondary school I wasn’t taught much about it. So, when I go to tertiary educational institutes like the university, it was like I sometimes don’t really know what to write for self-reflective essays." (S20)  "In Singapore I would have to say for the first few years in education until the junior colleges, there is not really any self-reflection elements at all until only at the university level. Only until then suddenly there is a lot of reflecting to do as required by the lecturers. Yeah, the earlier years of education could have better prepared us for that." (S29) |
|  |  | Low emphasis in the university | "It would be better if the university could place more emphasis on self-reflection. For example, such a study is helpful as an indication that self-reflection is a critical component in university education." (S16) |
|  | Social factors | Low peer encouragement | "Yeah, my friend thinks self-reflection is not very useful. Yeah...I think this also… influences me…" (S20) |
|  |  | Low family encouragement | "Because just talking to people, like if the parents are more educated, then naturally they will also impart reflection skills to the children. Yeah, on the other hand if people are not taught reflection since young by their parents, self-reflection will not come naturally to them when they reach the university." (S16) |
|  | Teacher factors | Lack of advocacy from teachers | "For me, if the lecturer never tells me to do, then I won’t do it. Yeah...I mean the lecturers must first recommend self-reflection to us... if not, I don’t think I would really use it." (S20)  "The professors or lecturers they should first support this – if I feel like they are the reflective type then I think it leaves a strong impression on the students and we will be more likely to follow suit too" (S16)  "And then there are some Profs that are more stoic, you know like a bit more serious, they just accept only certain answers, and they would not encourage you to share your thoughts or feelings freely, then of course I am not going to risk it to affect my grade." (S1) |
|  |  | Lack of guidance or feedback from teachers | "I think it is because I don’t really have a clear understanding of how self-reflection can be applied to public health. There are no clearer sets of instruction on how to do self-reflection, nor are there sufficient guidance or feedback from the lecturers or professors... Yeah. I think there is a lack of tangible support from them... I think the key issue is the lack of guidelines or frameworks to do self-reflection from the lecturers, because I tend to be very lost and confused, so I would need that." (S14)  "I think if there is more feedback or guidance from the lecturers, … it would be helpful because I need to know whether what I am doing is correct, if no one is there to guide me, I do not feel confident enough to reflect." (S33) |
|  |  | Imbalanced power relationship between teacher and student | " For me, I don’t feel comfortable in reflecting or revealing what I really think. Because I feel like my lecturer has some sort of authority over the content he or she is sharing. So, I feel it will be not very nice for me to challenge him or her, even though when I have my doubts or when I think otherwise." (S14)  "Hmm... like share my thoughts, is it? ...Then I wouldn’t be so willing to say what I truly think or feel... I tend to give politically correct answers...because this is safer, and I am not willing to challenge the lecturers because they know more than me." (S20)  "I tend to reflect based on what the lecturer wants...because they are more powerful than me and I won’t want to go against them." (S3) |
|  |  | Teachers did not incorporate it as part of the curriculum or assessment | "I mean self-reflection is not part of the module nor is it part of the assessment. So, I feel I shouldn’t be wasting time on it since it is not even part of the requirements to begin with, isn’t it?" (S3) |
|  |  | Nature of content taught | "Probably I think it is very reliant on what the content is about. Because modules that focuses on statistics. It is so mathematical and so like I guess that doesn’t require self-reflection." (S1) |
|  | Environment factors | Inadequate opportunities for self-reflection | "I think there needs to be opportunities for us to reflect in the first place, I don’t think I have ever had a module that asked for a reflection from me. Yeah so, there was like no formal space in the curriculum at all for this in the first place? Given this situation, even if I want to reflect, there are no opportunities for me to do so." (S41)  "Hmm, because I don’t think there are many activities on self-reflection that I need to share. Other than the group work that I have mentioned... I don’t see any of it at all." (S20)  "I haven’t been asked to share... there was only this peer review thing, which I don’t really consider it as self-reflection because it is more of like reflection on the group, or the team members, and does not involve myself at all. There’s no opportunities for us to do that." (S14) |
|  |  | Fear of being judged by others | "I fear of being judged; I think certain hostilities can be expressed through tones as well as choice of words. When that happens, then you just feel reluctant to share your thoughts openly." (S1)  "Because I do not feel safe to share how I think or feel with the general audience, perhaps it is fine only with my close friends. But not openly in the class where people could judge you based on what you say." (S14) |
|  |  | Lack of anonymous option | "Let’s say if the responses are not kept confidential or if there is no anonymity option, I am less keen to share, I prefer to be more discreet about my thoughts." (S3)  "Reflection to me is a very personal thing and if there is no privacy and personal identifiers are needed, then I might be less inclined to do it." (S1) |
| Barriers internal to student | Negative feelings associated with prior self-reflection | - | "Erm, I once get criticised for something when I have to share my self-reflection and the feeling is quite terrible." (S18)  "The last time when I self-reflect, I did not enjoy the process. I have to do it eventually to improve myself and not because I really like the feeling of it." (S14) |
|  | Low knowledge to perform self-reflection | - | "I don’t really do it because I don’t know how to do it. Yeah... I don’t know what questions to ask myself or what I should be doing." (S33) |
|  | Low self-efficacy to perform self-reflection | - | "I don’t think I have the confidence to reflect. Honestly, I don’t have much experience in reflecting and that is probably why I have no confidence." (S29) |
|  | Did not see the need for self-reflection | - | "Why? Because my personal view on self-reflection is not a good one. Because I really think it doesn’t help me in any sense, for me it will be quite a waste of time...Hmm… I think it is a personal belief that I don’t think self-reflection is useful to me." (S20)  " Yeah... I think like my personal belief is I am a very pragmatic person, so I feel for self-reflection, it can only apply to a very narrow range of modules, so personally for me I, I don’t see the need to do self-reflection." (S14) |
|  | Perception that self-reflection was too cumbersome and time consuming | - | "I guess it depends on the frequency also. Sometimes like having a reflection after every class, is a bit tedious also. Then after a while, I won’t do it anymore because too tedious for me." (S41)  "I think if reflection was applied to all public health modules and I was taking like 5 modules that semester, I would probably drown. Yeah...I mean like there are other commitments too. Sometimes you really need to set aside time, like set aside time and a space. But then more often than not, things just get clogged up in everywhere." (S1)  "I guess if I am very busy, then I wouldn’t find the time like to sit down and reflect."(S16)  "I will say lack of time and too much stuff on my plates to be able to reflect." (S29)  "I feel that writing all these reflective essays is very cumbersome, I will have to spend a lot of time on it and then instead of it being like an opportunity to reflect, it becomes more of like a chore. Yeah... as university students, we have like time constrains and if you compare us with other graduates in other countries, I will say that we don’t have much time."(S3) |
